# Automatic mental simulation in native and non-native speakers

**DOI:** 10.3758/s13421-024-01533-8

**Published:** 2024-02-14

**Authors:** Samuel J. A. van Zuijlen, Sharon Singh, Kevin Gunawan, Diane Pecher, René Zeelenberg

**Affiliations:** Erasmus University, Woudestein T13-31, PO Box 1738, 3000 DR Rotterdam, the Netherlands

**Keywords:** Mental simulation, Color match, Perceptual symbols system, Sentence-picture verification task, Language comprehension, Non-native speaker, Bilingualism

## Abstract

Pictures of objects are verified faster when they match the implied orientation, shape, and color in a sentence-picture verification task, suggesting that people mentally simulate these features during language comprehension. Previous studies had an unintended correlation between match status and the required response, which may have influenced participants’ responses by eliciting strategic use of this correlation. We removed this correlation by including color-matching filler trials and investigated if the color-match effect was still obtained. In both a native sample (Experiment 1) and a non-native sample (Experiment 2), we found strong evidence for a color-match advantage on median reaction time and error rates. Our results are consistent with the view that color is automatically simulated during language comprehension as predicted by the grounded cognition framework.

## Introduction

If we write our friend about the new car we bought, how does our friend know exactly what we mean when we say “car”? How is this meaning of a car represented in her mind when she comprehends language relating to an object? The world comprises many objects, and when we read or talk about them, we almost instantly understand what is meant and what the most salient features of the object are (given that we have experienced the object before). Because language comprehension plays such a big part in how humans come to understand and interact with other people, it is important to understand its mechanisms. A question that has interested cognitive scientists is whether perceptual features of objects are represented by readers when they comprehend language. According to grounded cognition theories, the features that are activated during language comprehension are based on earlier perceptual-motor experiences with the objects described in the sentences (Barsalou, 1999; Barsalou et al., 2003). On this account, people represent the meaning of language by mentally simulating the perceptual and motor processes that they would also have used if they were immersed in the real-world equivalent of what is described by the language. In the present study we investigated whether such mental simulations also underlie understanding of non-native languages.

Several studies testing native speakers have obtained evidence for visual mental simulations during language comprehension. When participants are given a verbal property verification task (e.g., “Is a banana yellow?”), their responses are influenced by visual characteristics of the properties (Borghi, 2004; Borghi, et al., 2004; Morey et al., 2021; Solomon & Barsalou, 2004; Spivey & Geng, 2001; Taylor & Zwaan, 2008; Zwaan & Taylor, 2006) and trial-to-trial switches in perceptual modality (Ambrosi et al., 2011; Connell & Lynott, 2011; Marques, 2006, Pecher et al., 2003, 2004; Van Dantzig et al., 2008; Vermeulen et al., 2007). One of the earliest studies on mental simulations during language comprehension found that participants were faster and more accurate in verifying that a pictured object (e.g., an upright nail) was mentioned after reading a sentence implying the depicted orientation (e.g., “He hammered the nail into the floor”) than after reading a sentence implying a different orientation (e.g., “He hammered the nail into the wall”) (Stanfield & Zwaan, 2001). In this so-called sentence-picture verification task, participants decide whether the object presented immediately after the sentence was mentioned in the preceding sentence or not. It seems that participants mentally simulate the content of the sentence, and that subsequent verification of the depicted object is faster and more accurate when the visual feature implied by the sentence matches that of the picture even though that feature was not explicitly mentioned. Match effects have now been obtained for various sensory features such as shape, distance, or size (De Koning et al., 2017; Pecher, van Dantzig, Zwaan, et al., 2009; Sato et al., 2013; Winter & Bergen, 2012; Zwaan et al., 2004; Zwaan & Pecher, 2012; Zwaan et al., 2002; Zwaan et al., 2018). Note that the simulation account of the match effect differs from mental imagery, which is largely conscious and concerns itself with imagination. Mental simulation is assumed to be unconscious, and is the underlying process of conceptual processing (Pecher, van Dantzig, & Schifferstein, 2009a, 2009b; Solomon & Barsalou, 2004; Vermeulen et al., 2008; Zwaan & Pecher, 2012).

Relatively little attention has been devoted to the role of mental simulations in language comprehension of non-native speakers. Some researchers have argued that mental simulations may be less vivid when people read a non-native language. Especially for a second language that is learned later in life, in a formal setting such as school, people may have weaker links between language and sensory experiences (Foroni, 2015; Kogan et al., 2020; Norman & Peleg, 2022). The strength of mental simulations may depend on proficiency in the second language (e.g., Dijkstra & van Heuven, 2002; Monaco et al., 2019; van Heuven & Dijkstra, 2010; Zhao et al., 2019; but see Bergen et al., 2010). Empirical evidence for mental simulation in non-native speakers is relatively sparse and seems to come mainly from paradigms that aim to assess involvement of the motor system. There is some evidence that non-native speakers perform mental simulations (Dudschig et al., 2014; Wheeler & Stojanovic, 2006), although this may depend on the extent to which a person’s native language can be mapped onto the meanings of their non-native language (Ahlberg et al., 2018). In sentence-picture verification tasks the evidence for mental simulations in non-native speakers is weak at best. Chen et al. (2020) presented items that matched or mismatched in implied shape in a delayed recognition task (modelled after a study with native speakers by Pecher, van Dantzig, Zwaan, et al., 2009) to participants who were native speakers of Cantonese and non-native speakers of English and Mandarin. They found a match effect in reaction times only when sentences had been read in the participants’ native language and not in either of the two non-native languages. Norman and Peleg (2022) found a shape-match effect for native Hebrew speakers when sentences were in Hebrew but not when sentences were in their non-native language English. Ahn and Jiang (2018), on the other hand, did obtain similar shape and orientation match effects for native and non-native speakers of Korean. However, their study used different sentences in the match and mismatch conditions, which introduced a confound between condition and stimulus materials, raising questions about the validity of the results.

In the present study we investigated the mental simulation of color using the sentence-picture verification task in native and non-native speakers of English. Objects (e.g., a leaf) can take different colors (green, brown), which can be implied by a sentence (“The leaf was on the tree” vs. “The leaf was on the ground”). Although initially a mismatch advantage was obtained (Connell, 2005; 2007), later studies, using larger samples, did not report this color-mismatch advantage (Hoeben-Mannaert et al., 2017; Zwaan & Pecher, 2012). Rather, both studies reported a positive match effect of color (i.e., a match advantage), where images that matched the preceding sentence on the object and color produced faster responses than images that mismatched the color (also see De Koning et al., 2017). Together, the results across different studies suggest that people represent color during language comprehension.

Before testing whether match effects can be found for non-native speakers, we wanted to improve the paradigm by eliminating the potential for strategic responding. If sensory simulation is an integral part of language comprehension, simulations should be automatic whenever language comprehenders process the meaning of a sentence. A noticeable feature of the sentence-picture verification task is that there is a correlation between the match status (match vs. mismatch) and the required (i.e., correct) response (‘’yes’’ vs. ‘’no’’). Consider, to make this more concrete, studies that have investigated the color-match effect. Participants in these experiments read a sentence that is followed by an object picture and decide whether the object is mentioned in the preceding sentence. On critical trials (i.e., where the depicted object was mentioned in the preceding sentence), typically half consist of a color-match trial and half consist of a color mismatch trial. On filler trials (i.e., where the depicted object was not mentioned in the preceding sentence, thus requiring a “no” response), color match is not controlled or manipulated. Because objects can have many different colors this results in few, if any, filler trials on which the object color matches the color implied by the sentence. Consequently, the color match/mismatch status is correlated with the required response. If the color of the object in the picture matches that of the implied color in the sentence, there is a high probability that the object was mentioned in the sentence.^1^ On the other hand, if the color of the object in the picture does not match the implied color in the sentence, the probability that the object was mentioned in the sentence is well below 50%.^2^ If participants pick-up on this correlation they may use it to aid their responses in the sentence-picture verification task.

Ample research, using a variety of tasks, stimuli, and procedures, has shown that people are sensitive to correlations between stimuli as well as correlations between stimuli and responses (e.g., Garcia et al., 1955; Parise et al., 2012; Reber, 1967; Zeelenberg et al., 2004), even without explicit instructions to detect, learn, or use such correlations to facilitate responding. As an example, consider a well-known study by Neely et al. (1989), who investigated semantic priming effects in a lexical decision task. In a lexical decision task, participants make binary decisions about the lexical status (word vs. nonword) of the target stimulus. A characteristic of the primed lexical decision task is that there is a correlation between the relatedness of the prime and the target and the required response. If the prime and target are semantically related (e.g., *cat* – *dog*), the target is a word, because nonwords are not semantically related to words. Neely et al. assumed that participants use this correlation in the decision process. Participants will be biased to give a “word” response if they detect a relation between prime and target and they will be biased to give a “nonword” response if they do not detect a relation between prime and target. By manipulating the nonword ratio, Neely et al. showed that participants are sensitive to the correlation between relatedness of the prime and target and the lexical status of the target. The nonword ratio is the probability that the target is a nonword given that it is unrelated to the prime. If the nonword ratio is high, the absence of a relation between prime and target is highly predictive for the fact that the target is a nonword. If the nonword ratio is low, however, the absence of a relation between prime and target is less informative. Neely et al. found larger priming effects with high nonword ratios, indicating that participants are indeed sensitive to the correlation between the relatedness of the prime and target and the lexical status of the target stimulus. In addition to these more strategic decisional processes that are biased by this correlation and contribute to the semantic priming effect, researchers have argued that semantic priming is also due to automatic activation processes (e.g., den Heyer et al., 1983; McNamara, 1992; Neely, 1977; Neely et al., 1989). Because strategic processes may have the same effects on performance measures as automatic processes, it is difficult to conclude that the observed priming effects are due to automatic processes. Therefore, researchers have designed experiments that aim to eliminate the contribution of strategic processes. Consistent with an automatic activation view, semantic priming effects are also obtained when the contribution of strategic processes is prevented (e.g., Balota & Lorch, 1986; de Groot, 1983; Pecher et al., 2002). Thus, to investigate automatic conceptual processes, it is important to use procedures that eliminate more strategic contributions to an effect.

In the present study, we investigated if a color-match effect in the sentence-picture verification task is also found when the correlation between the presence of a color match and the required response is eliminated. Thus, in contrast to previous studies on the match effect, the presence of a match was not *predictive* of the required response. Crucially, we added color-match filler (i.e., “no”) trials to the stimuli presented in the experiment. That is, even on trials where the object was not mentioned in the preceding sentence, the object color could still match the implied color of the object mentioned in the sentence (e.g., the sentence implies pink paint and a picture of a pink marshmallow is shown). In doing so, we removed the correlation between color match and the required response. A match effect in the absence of this correlation provides stronger evidence that match effects are not dependent on mental simulations that are strategically employed in the sentence-picture verification task. In our study, the presence of a color match does not inform participants about the required response. If language comprehenders, given that they process the sentence at a semantic level, automatically simulate properties such as the color of an object mentioned in a sentence, we should still obtain a color-match advantage. If, on the other hand, the color-match advantage depends on strategically employed mental simulations to aid responding in the sentence-picture verification task, no such advantage would be present because there is no correlation between color match and the required response. If under these circumstances we still find a match effect for native speakers in Experiment 1, we will then proceed to test a sample of non-native speakers in Experiment 2 using the same stimulus materials.

## Experiment 1

### Method

#### Preregistration

Predictions, method (including exclusion criteria), and planned data analyses of Experiment 1 were preregistered on the Open Science Framework (OSF) in advance of data collection (https://osf.io/r6b7j/).

#### Participants

A total of 371 native speakers of English were recruited for the study. The data of 300 participants were included in the final analysis (detailed information about participant exclusion is provided later). The mean reported age was 31.0 (range 18–73) years, 165 participants reported being female. They reported their country of birth/country of residence as the UK (51.4%/57.8%), South Africa (17.3%/18.1%), USA (6.8%/8.4%), Australia (3.0%/3.5%), Ireland (3.0%/3.0%), Canada (2.4%/3.0%), Germany (1.1%/0%), and 26 other countries or did not provide this information (14.3%/6.2%). Participants were recruited on Prolific and received £1.25 for their participation. Completing the experiment took approximately 8 min. The posting on Prolific was offered only to native speakers of English. Based on the effect size reported by Hoeben-Mannaert et al. (2017) for their Experiment 1 (*d* = 0.26), we computed the required sample size to obtain a statistical power of .95 with a two-tailed paired-sample *t*-test (α = .05) using G*power (Faul et al., 2009). The required sample size amounted to 195 participants. To be on the safe side, we decided to test 300 participants. We included only participants who met all the following criteria: (1) participants completed the experiment, (2) participants indicated that they were native speakers of English, (3) participants responded correctly on at least 80% of the sentence-picture verification trials, and (4) participants responded correctly to at least 50% of the sentence comprehension questions. The data from participants who failed to meet one or more of these criteria were excluded from the analyses. Removed participants were replaced by new ones who were tested with the same counterbalancing version.

#### Stimulus materials and software application

The present experiment used the same critical stimuli as Hoeben-Mannaert et al. (2017). These consisted of 16 sentence pairs and 16 picture pairs. The two versions of a sentence pair, each one implying a different color, could be coupled with the two versions of a picture pair, each one in a different color, to form matching or mismatching trials. Across four list versions, for each of the 16 critical objects, one of the sentences in a pair was coupled with one of the pictures in a pair, resulting in four different combinations of sentence-picture pairs (see Table 1 for examples). Thus, each participant saw only one sentence and one picture of an object. In this manner, four counterbalanced versions were created so that, across participants, each sentence and each picture were presented equally often in the color-match and color-mismatch condition. The same set of 16 filler items was presented to all participants. Each counterbalanced version thus included 16 critical sentences paired with 16 critical pictures (i.e., eight color-match trials and eight non-match trials) and 16 sentence-picture pairs (also eight color-match trials and eight non-match trials) that were used as fillers. As shown in Table 1, on filler trials the object color matched or mismatched the implied color of the sentence, but the depicted object was not mentioned in the preceding sentence. Half of the filler trials were followed by a comprehension question with an equal number of ”yes” and ”no” responses (see Table 1), to ensure that participants did not merely skim the sentences. An additional set of eight sentence-picture pairs and eight comprehension questions was used for practice. We used the same practice pairs for all participants.

**Table 1:**
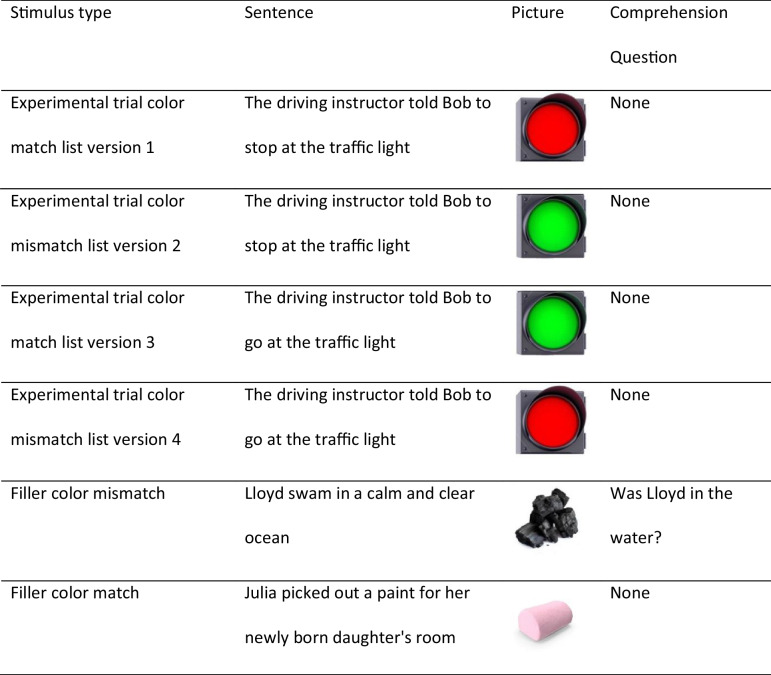
Example of experimental and filler stimuli

The experiment was programmed in Inquisit (https://www.millisecond.com/), a software application developed for online psychological testing. All pictures were royalty-free images obtained through the Google images search engine. All images were of an object in one dominant color against a neutral background. We only selected images of objects that have limited color variations (e.g., a ripe vs. an unripe tomato). Image height was 50% of the screen. See Online Supplementary Materials (OSM) for examples of pictures. All text was presented in the letter font Verdana (letter height 3% of the screen) against a white background.

#### Procedure

**Fig. 1:**
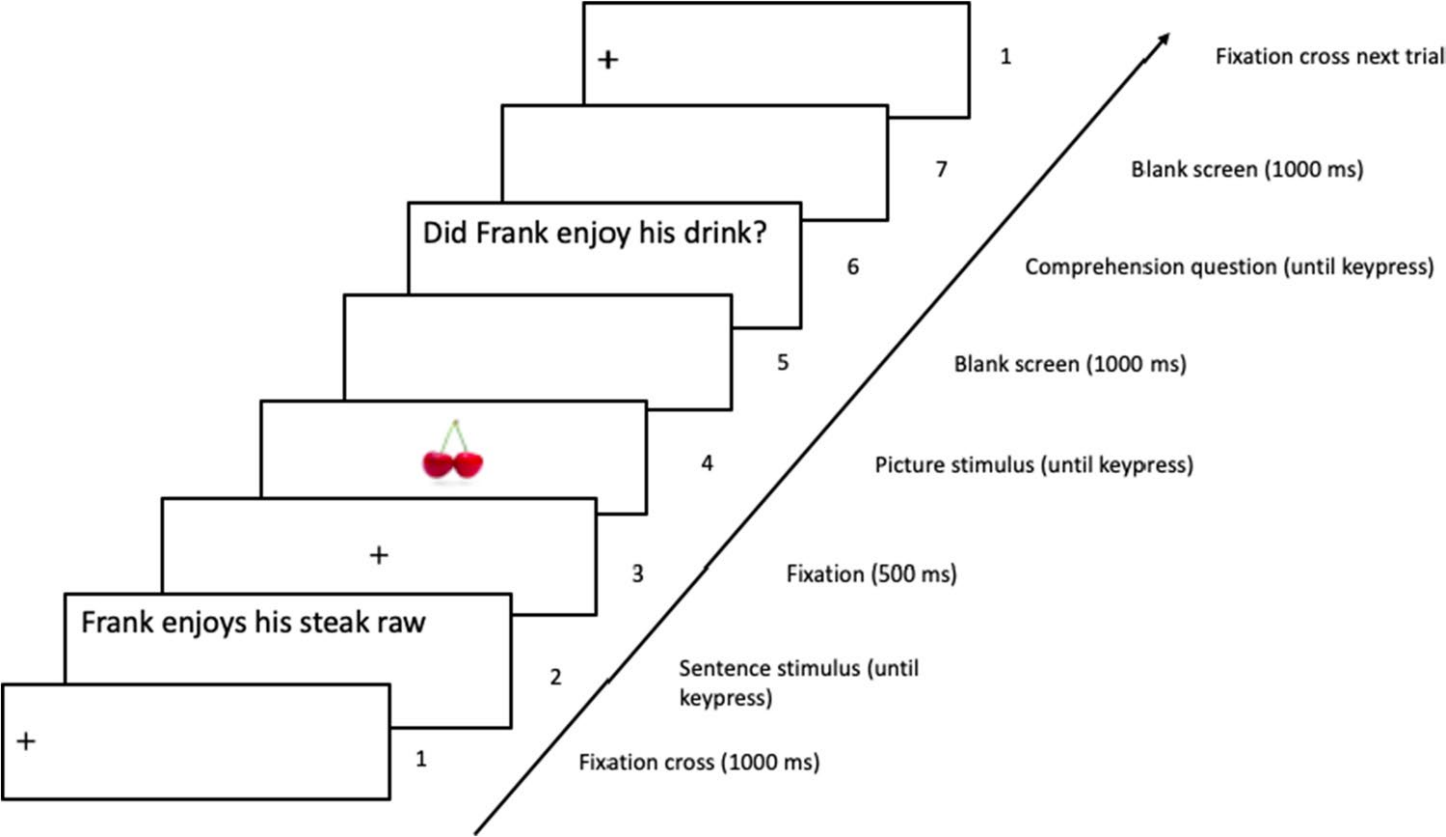
Example color-match filler trial and comprehension question. If the participants understood the sentence, they pressed the spacebar to continue. Only panels 1–5 were shown on experimental trials, and on filler trials without comprehension question

Participants selected the study on Prolific and started the experiment from their personal computer or laptop. Upon opening the experiment, participants gave informed consent and read a welcome text and instructions. Participants were instructed to respond as quickly and as accurately as possible. They responded “yes” (by pressing the M key on the keyboard) when the depicted object was mentioned in the preceding sentence. They responded “no” (by pressing the Z key on the keyboard) when the depicted object was not mentioned in the sentence. Each trial started with a fixation cross (+) presented for 1,000 ms vertically in the middle of the screen and horizontally aligned to the left, where the first character of the sentence would appear (see Fig. 1). The fixation cross was followed by a sentence. After the sentence was read and understood, the participant pressed the spacebar to proceed. Another fixation cross (+) was presented centrally for 500 ms. Following this fixation cross, an object picture was presented centrally to which participants responded “yes” or “no” using the M or Z key, respectively. On half of the filler trials the response to the picture was followed by a comprehension question. Comprehensions questions required a “yes” (M key) or “no” (Z key) response. If participants made an error on picture trials or comprehension questions, the feedback message “Incorrect” was presented for 500 ms. A 1,000-ms intertrial interval followed the response of the participant (or feedback in case of an incorrect response). Trials were presented in a random order. Different random orders were generated for each participant.

Participants first completed eight practice trials followed by 32 experimental trials (16 of which required a “yes” response and 16 of which required a “no” response) presented in random order. The trial procedure was the same for practice and experimental trials. For both practice trials and experimental trials, half consisted of color-match trials and half consisted of color-mismatch trials.

The experiment ended with a closing questionnaire that asked participants for their native language, gender, and age, followed by a final thank you message.

#### Data analysis

Following previous studies (Hoeben-Mannaert et al., 2017; Stanfield & Zwaan, 2001; Zwaan & Pecher, 2012; Zwaan et al., 2002) and our preregistered analysis plan, statistical analyses were based on the median reaction times. For each participant and condition the median reaction time for correct responses was determined and entered in the analyses. We conducted a paired-samples *t*-test to compare the median reaction times in the color match and color mismatch conditions (using α = .05). A comparable analysis was performed on the mean error rates.

### Results and discussion

Based on our preregistered criteria, the data from 71 participants were excluded. Thirty-three participants indicated that their native language was one other than English. Twelve participants did not finish the experiment. Moreover, we removed the data from one participant due to a low comprehension score (below 50%),^3^ and we removed the data from ten participants due to a low overall accuracy on the critical trials (below 80%). Finally, we removed the data from 15 participants to ensure an equal number of participants in each counterbalancing version.^4^ We analyzed and report the data of the remaining 300 participants (56% female, mean age = 30.8 years, *SD* = 11.7). The data of all experiments reported in this article are available at https://osf.io/r6b7j/.

**Fig. 2:**
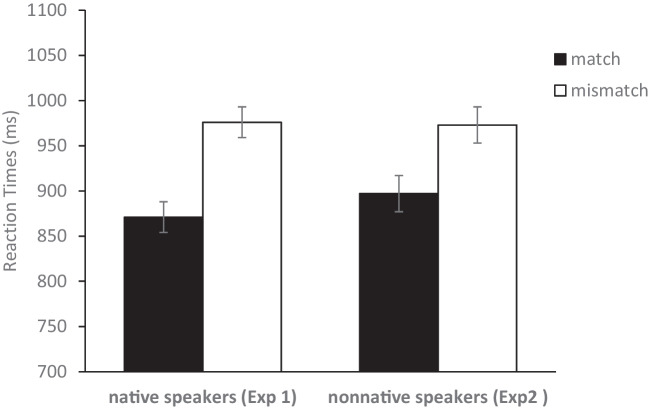
Mean median reaction times for match and mismatch conditions in Experiments 1 and 2. In both experiments sentences were presented in English. Error bars represent standard error of the mean difference between the match and mismatch conditions (Loftus & Masson, 1994)

The analyses were based on only the critical trials. Figure 2 shows the mean median reaction times (RTs) for the match and mismatch condition (only trials with a correct response were included in the analyses). Participants responded faster on match trials than on mismatch trials. The 106-ms color-match advantage (871 ms vs. 976 ms) was significant, *t*(299) = 6.23, *p* < .001, *d* = 0.36. Moreover, participants made fewer errors on color-match trials than on color-mismatch trials (4.3% vs. 13.1%), *t*(299) = 10.55, *p* < .001, *d* = 0.61. Thus, we found a color-match advantage on RTs and error rates even when the color match status was uncorrelated to the required response. These results are consistent with the view that native speakers of English mentally simulated color during language comprehension.

Our results are in line with previous studies reporting a color-match advantage (De Koning et al., 2017; Hoeben-Mannaert et al., 2017; Zwaan & Pecher, 2012). Our evidence in favor of a match advantage, however, stands in contrast with studies reporting a mismatch advantage (Connell, 2005; 2007). We are not aware of a good explanation for this discrepancy in results. However, several researchers have now obtained a color-match advantage in preregistered experiments (Hoeben-Mannaert et al., 2017; the current Experiment 1). Moreover, the positive color-match effect aligns with similar findings for shape (e.g., Zwaan et al., 2002, 2018; Zwaan & Pecher, 2012) and orientation (e.g., Stanfield & Zwaan, 2001; Zwaan & Pecher, 2012). We are not aware of anyone reporting a negative match effect for implied shape and orientation. Thus, it seems that native speakers mentally simulate the color of an object when comprehending language.

Experiment 1 provided additional evidence for the idea that simulations are an automatic consequence of sentence processing. In Experiment 2 we asked whether evidence for mental simulations is also found when language comprehenders read sentences in their non-native language. Given the mixed results for non-native speakers in previous studies using sentence-picture verification tasks (Ahn & Jiang, 2018; Chen et al., 2020; Norman & Peleg, 2022), we investigated if the color-match effect that we observed in Experiment 1 with native speakers would be found in a group of non-native participants. If non-native speakers of English, like native speakers, use a similar visual simulation process during language comprehension, we should find a color-match effect as we did in Experiment 1.

## Experiment 2

### Method

#### Participants

Participants were non-native speakers of English who studied Psychology at Erasmus University Rotterdam in the Netherlands. They received course credits for their participation. We tested students at our own university because we know they are highly proficient non-native speakers of English. Erasmus University offers a Dutch language track and an international (English language) track for the 3-year Psychology bachelor program. The main difference between these tracks is in the small group meetings that students typically attend two or three times a week to discuss the literature and work on exercises. Small group meetings are either in Dutch or English, depending on the language track. For both tracks, plenary lectures are given in English and the literature consists of texts written in English (mainly textbooks and journal articles).

Before enrolling at the university, most Dutch students have had 6 years of secondary education throughout which they have taken English classes. These students are frequently exposed to English outside of formal educational settings as most movies and television programs in the Netherlands are shown in their original format (with Dutch subtitles for broadcasts in a language other than Dutch). Many Dutch people frequently travel to other countries where they often speak English. In addition, English language songs are very popular in the Netherlands. The educational and cultural background of our international students is more diverse. However, all international students self-selected to take part in the English language program at Erasmus University and provided proof of English proficiency prior to admission – for example, by having obtained a secondary education diploma of which English was part of the final exam, by having attended secondary school in an English-speaking country (e.g., UK), or having obtained a TOEFL (Test of English as a Foreign Language) score ≥ 6.5 or IELTS (International English Language Testing System) score ≥ 90.

The sample included a wide variety in native languages; together the 200 participants listed 24 different native languages. Most prevalent were native speakers of Dutch (125 participants). Other languages that were listed as a native language by four or more participants were German (15 participants), Russian (six participants), Arabic (five participants), Turkish (five participants), Italian (five participants), Polish (four participants), Romanian (four participants), Spanish (four participants), and Vietnamese (four participants).

Although we initially planned to analyze data of 300 participants, we eventually decided to include 200 participants in the analyses because the recruitment of participants proceeded slower than we had hoped. Although we anticipated that the effect size in Experiment 2 might be somewhat smaller than the one we obtained in Experiment 1 (*d* = 0.36), because we tested non-native speakers, we expected that 200 participants would be sufficient to ensure a high statistical power (as indicated by the initial power analysis for Experiment 1). We collected data from 17 May 2021 until 19 March 2022.

#### Procedure

The procedure was identical to that of Experiment 1.

#### Data analysis

The data analysis was the same as that of Experiment 1.

### Results and discussion

The data of 56 participants were excluded based on our criteria and we analyzed data of 200 participants (80% female, mean age = 21.3 years, *SD* = 3.8). Eleven participants indicated English as their native language and their data were consequently removed. Five participants did not finish the experiment. We removed the data of three participants due to a low comprehension score (below 50%),^5^ and we removed the data of ten participants due to a low overall accuracy on the critical trials (below 80%). Finally, we removed the data of 27 participants to ensure an equal number of participants (i.e., 50) in each counterbalancing version.

The analyses were based on only the critical trials. Figure 2 shows the mean median RT for correct responses in the match and mismatch condition. Participants responded faster on match trials than on mismatch trials. The 76-ms match advantage (897 ms vs. 973 ms) was significant, *t*(199) = 3.81, *p* < .001, *d* = 0.27. Participants also made fewer errors on match trials than on mismatch trials (5.2% vs. 17.9%), *t*(199) = 10.33, *p* < .001, *d* = 0.73. Thus, we obtained a color-match advantage on median RT and mean error rates even when the correct response was uncorrelated to the color match. The results indicate that non-native speakers of English (at least from the population we sampled) mentally simulated color during language comprehension.

#### Exploratory analyses

To investigate if the size of the match effect was different for native and non-native speakers, we performed an additional ANOVA on the combined data from Experiments 1 and 2. For RTs we found no interaction between experiment and match effect, *F*(1,498) = 1.25, *p* = .264, partial *η*^2^ = .00, indicating no difference in the size of the match effect between the native and non-native speakers. For error rates we, somewhat surprisingly, found a larger match effect for non-native than for native speakers, *F*(1,498) = 7.62, *p* < .01, partial *η*^2^ = .02. The interaction for error rates should be interpreted with caution because it was accompanied by a nonsignificant trend in the opposite direction for RTs. Thus, our results provide no evidence that the match effect was smaller for non-native than native speakers.

## General discussion

Previous studies reported findings consistent with the idea that people mentally simulate features such as shape, orientation, and color during native language comprehension (e.g., De Koning et al., 2017; Hoeben-Mannaert et al., 2017; Stanfield & Zwaan, 2001; Zwaan et al., 2002; Zwaan et al., 2018; Zwaan & Pecher, 2012). Crucially, on critical trials the depicted object could match or mismatch the object in the sentence on a feature such as shape, orientation, or color that was implied by the sentence but not explicitly mentioned. Studies using the sentence-picture verification task have shown that participants respond faster on match trials than mismatch trials, bolstering the view that people perform mental simulations during language comprehension. However, a common property of these studies was that they did not control the match status for filler trials. Consequently, in the typical sentence-picture verification experiment there is a correlation between the match status and required response. Participants may strategically use such correlations to aid task performance (cf. Neely et al., 1989). By including color-match filler trials in our study, we removed the correlation between color match and the required response. In Experiment 1, we observed that even though color match was uncorrelated to the required response, participants still verified color-match trials faster and with greater accuracy than color-mismatch trials. This is consistent with the idea that, in this task, color simulations are automatic and not dependent on strategies elicited by task demands. These results strengthen the claim that participants mentally simulated an object’s color while processing the meaning of a sentence in their native language, regardless of the specific task demands. This finding aligns with previous research that suggested that mental simulations are automatic and outside of awareness (Barsalou, 1999; Pecher et al., 2009a, 2009b; Zwaan & Pecher, 2022), at least when sentences are processed at a semantic level. Language comprehenders use previous perceptual-motor experiences to construct simulations of objects during language comprehension as proposed by Barsalou’s (1999) perceptual symbols system theory.

In Experiment 2 we extended the match advantage to non-native speakers. Previous studies investigating mental simulations in non-native speakers have found inconsistent results. Whereas some studies found evidence for mental simulations in non-native speakers (Dudschig et al., 2014; Wheeler & Stojanovic, 2006), other studies, in particular those using the sentence-picture verifications task, did not find such evidence (Chen et al., 2020; Norman & Peleg, 2022). In contrast, we obtained a clear match effect, which indicates that the non-native speakers from our pool adopt a native-like comprehension style.

Our results suggest that, at least for the proficient non-native speakers that we tested, language comprehension processes in the native and non-native language are very similar. It seems that non-native speakers mentally simulate the content of sentences during language comprehension. Thus, even though the non-native language is acquired at a later age than the native language, comprehension in the non-native language seems to rely on sensorimotor information just like comprehension in the native language. On the one hand one might argue that this is perhaps not surprising given that the lexical representations of the native and non-native language are connected to a shared conceptual system (Kroll & Stewart, 1994; Potter et al., 1984; Zeelenberg & Pecher, 2003). The lexical representations of the native and non-native language are often assumed to activate the same or at least very similar conceptual information. On the other hand, it has been argued that because the non-native language is learned in a different way than the native language, sensorimotor information might not play a role during non-native language comprehension (Foroni, 2015; Kogan et al., 2020). The reasoning is that because the non-native language is learned in a formal setting in which new words are not learned through bodily experiences and direct interaction with the physical world, the connections between the lexical representations and sensorimotor information are much weaker than for the native language. The present results seem to be at odds with this view; the mental simulation of visual characteristics seem to be a part of the language comprehension processes in the native and non-native language. Of course, this issue merits further investigation to see whether this holds for visual characteristics other than color and whether this also holds for non-native speakers who are less proficient or do not use their non-native language as frequently as our participants.

We can only speculate as to why some studies have failed to find evidence for mental simulations in non-native speakers. One factor may be related to statistical power of some published studies. Recent studies that investigated the color-match effect in native speakers had sample sizes of around 200 (Hoeben-Mannaert et al., 2017; Zwaan & Pecher, 2012), which is an adequate size according to our power analysis. In Experiment 2, we therefore tested 200 participants, which is much larger than samples used in previous studies with non-native speakers. For example, Chen et al. (2020) had a sample size of 36 participants and Norman and Peleg (2022) had a sample size of 80. While they did find a shape-match effect in the sentence-picture verification task for native speakers, they did not find a match effect for non-native speakers. Possibly, the power to find a potentially smaller effect for non-natives was low.

A second factor may be language proficiency. We did not specifically measure language proficiency, but all participants were university students in a program that requires participants to be proficient in English as the lectures and the course literature are in English. Students can enroll only if they can demonstrate proficiency in English. Moreover, proficiency in English is not only a requirement to enter the bachelor program at our university, participating in the program also means that students are frequently exposed to English (and are very likely to have been recently exposed to English prior to participating in Experiment 2). Although language proficiency, frequency, and recency of language use are all correlated to each other, they are not the same. For example, a Dutch person who resides in the Netherlands may be proficient in French but not be exposed much to French during day-to-day life. Such a person may have learned French during secondary education and maintain relatively high French-language proficiency during one or two trips a year to France. The extent to which such a person would show evidence of mental simulation when reading French sentences may depend on the recency with which the person has used French. Participants in our study process English language on an almost daily basis and this may have contributed to our finding of a match effect in non-native speakers of English. The participants tested by Chen et al. (2020) and Norman and Peleg (2022) resided in their home country at the time of participating in their studies and, consequently, may have used their non-native language less frequently than the participants in our sample. This difference may explain why we obtained evidence for visual simulations in non-native speakers and they did not. It would be interesting for future studies to investigate which language characteristics of non-native speakers, such as immersion (day-to-day exposure to and use of non-native language), initial age of learning the non-native language and manner of language acquisition, are related to the match effect.

Apart from statistical reasons and individual differences related to language proficiency and language use, results may also depend on specific tasks and procedures used to study mental simulations in non-native speakers. Chen et al. (2020) used a delayed version of the sentence-picture verification task in which all sentences were presented in a first phase and participants responded to pictures only after all sentences had been read. Although Pecher, van Dantzig, Zwaan, et al. (2009) found evidence for mental simulations in such a delayed task with native speakers, it is possible that the effects are short-lived for non-native speakers. Future studies could systematically investigate factors such as language proficiency and delay of testing to determine if and how these factors contribute to match effects in non-native speakers.

We used two online experiments. Although online testing may introduce more variability because participants use different devices and may be in a noisy environment, it lessens the influence researchers can wield over participants in terms of expectancies or unconscious experimenter effects. Moreover, it allowed us to recruit a more diverse sample of English-speaking adults in Experiment 1. Because participation was not limited to university students, the sample included a large variation in terms of age and educational background, a larger proportion of male participants than our sample of university students in Experiment 2, and participants that resided in different countries (primarily Australia, Canada, Ireland, South Africa, the UK, and the USA). The sample in Experiment 2 consisted of university students, and included participants who were native speakers of a large variety of different languages (e.g., Dutch, German, Russian, Arabic, Turkish, Italian, Polish, Romanian, Spanish, and Vietnamese). Together, these findings suggest that our effects are not limited to people from a relatively limited population with specific characteristics. Rather, our findings are consistent with the idea that mental simulations are automatically recruited and an integral part of the language comprehension process, even for non-native speakers.

## Footnotes

1 This probability is 100% if none of the filler trials are color-match trials.

2 This probability is 33% in the following typical case. Half of the trials consist of ‘’yes’’ trials and half consist of ‘’no’’ trials; half of the ‘’yes’’ trials are match trials and half are mismatch trials; for ‘’no’’ trials, none of the trials are match trials.

3 Note that the comprehension questions were merely added to stimulate processing the meaning of the sentence and not to select participants. Of the remaining participants, 95% made two or fewer errors. The overall accuracy for these participants on the comprehension questions was 89%.

4 In accordance with our preregistration, we retained the first 75 participants with valid data for each counterbalancing version and included these in the data analyses. The data of the remaining (later tested) participants were not included in the analyses.

5 Of the remaining participants, 88% made two or fewer errors on the comprehension questions. The overall accuracy for these participants on the comprehension questions was 83%.
